# Retraction: Treatment of Left Ventricular Circulation Disorder: Application of Echocardiography Information Data Monitoring

**DOI:** 10.2196/98672

**Published:** 2026-04-28

**Authors:** JMIR Publications Editorial Office

**Affiliations:** 1 JMIR Publications Toronto, ON

The JMIR Publications Editorial Office is retracting the article:

Treatment of Left Ventricular Circulation Disorder: Application of Echocardiography Information Data Monitoring [1]

This action is being taken due to identified concerns regarding potential manipulation of the submission process and authorship, the source of the animals used in the in vivo experiments, and the source of the study’s ethics approval.

The concerns with this publication were identified during an investigation of a series of articles with related concerns. The authors were contacted and offered an opportunity to comment on these findings and the proposed retraction but did not respond to any attempts at communication.
